# On becoming “essential”: Coronavirus lessons of ontology- from the migrant farmworker and us who consume the fruits of her labor

**DOI:** 10.1177/1473325020973340

**Published:** 2021-03

**Authors:** Odessa Gonzalez Benson

**Affiliations:** U-M School of Social Work, University of Michigan, Ann Arbor, MI, USA;

**Keywords:** epistemology, immigrant, ontology, migration, immigration, migrant labor, consumption, ontological turn

## Abstract

During the coronavirus pandemic of 2020, the migrant farmworker came to be deemed as ‘essential’ worker, thereby complicating discourses of ‘illegality’. This moment presents an ontological paradox, allowing means for examining the simultaneity of discursivity and materiality within the migrant farmworker as subject. Reflecting upon the ‘ontological turn,’ this article presents four lessons of ontology: post-representation, a focus on objects/artifacts, posthumanism and the politicizing of ontology, gained from interrogating the unraveling of discourse about the migrant-other in the time of coronavirus. These lessons coalesce in the entwining of the powers of discourse with the presence of the tangible, to understand the profound contradictions between a social structure that doesn’t want the migrant as neighbor and a capitalist economy that needs her as laborer. In closing, the article considers how shifting the gaze inward as consumers and then outwards unto our relationality can be a political act.

In a *New York Times* article on the coronavirus crisis of 2020, the headline stated that “Farmworkers, Mostly Undocumented, Become ‘Essential’ During Pandemic” (Jordan, 2020). The article continued as follows: the “coronavirus pandemic has brought an unusual kind of recognition: (the migrant’s) job as a field worker has been deemed by the federal government as ‘essential’ to the country.” This seems to present an ontological paradox. The “essentiality”^1^ of migrant farmworkers is central here, but that denotes a ‘being’, rather than a “recognition” to be “deemed” in the first place, as the news reporter wrote. Or does it?

As the coronavirus pandemic heightened our anxiety over food and consumption (as well as health and the economy), it also heightened awareness about the agriculture industry’s migrant farmworker who labors to produce our food.^2^ The large majority of all U.S. farmworkers are migrants, mostly from Mexico and South and Central America and about half are undocumented (U.S. Department of Agriculture, referenced in Jordan, 2020). The U.S. government issued letters to legally document each migrant farmworker, including undocumented migrants, as essential workers, letters they were to carry at all times to make them ineligible for deportation (Jordan, 2020). Further, these migrant workers were solely exempted from border restrictions and allowed entry into the United States (also into Canada, the United Kingdom, and other countries) (Bensadoun, 2020; O’Carroll, 2020).

The pandemic gives us the rare occasion to come face to face with a fundamental contradiction of our capitalist society: our demand for migrant labor and inclusion of migrants in our economic sphere but exclusion of migrants in our social sphere as neighbors (De Genova, 2013). The balance between supply and demand—delicate, teetering, and dubious—comes into full view. This irony of our capitalist means of production-consumption (Sassen, 1990) is laid bare by the pandemic, as are the inequalities inherent in our institutions and ways of life. This, particularly in our need for food as basic sustenance (as well as health care), has brought us all, quite suddenly, down to the base of Maslow’s pyramid. But, importantly, it is not only about food-as-product but also about people-as-producers: the migrant farmworker and, moreover, those of us who consume the fruits of her labor. To fully understand and then act upon these shifts and what the coronavirus pandemic reveals for us, needed are reflections on ontology.

## Turning to ontology

Over the last decade, academic fields have grappled with the “ontological turn,” from cultural anthropology to technology studies. Social work scholars too have discussed ontology (see Hardy, 2014; Houston, 2010; Webb, 2019), a discussion that could benefit from deeper engagement. The ontological is the latest turn in a long, historical, philosophical journey with “turns, turnoffs and roundabouts” (Sismondo, 2015) about “what is real” and “what can be known” (Staller, 2013), following the ‘discursive turn’ most recently. While a full elaboration of this journey is beyond the scope of this paper, I will provide a basic outline. Positivist ontology, as dominant in an “enlightened” science including the social sciences, posits a singular reality that can be fully observed and understood. However, critics have questioned how the observer/researcher is necessarily implicated in the observation, thereby calling into question the absoluteness and the realness that is presupposed (Staller, 2013). Critiques have thus over time led to post-positivist, constructionist, post-structuralist perspectives: seeing the unseen powers of discourse, language and interaction (via deconstruction, discursivity, or social constructionism), and experience (via phenomenology), and how they, too, construct realities and subjectivities (Feely, 2016; Zembylas, 2017). Representation or symbolic meaning, and the violent construction of such meaning, are foregrounded.

The ontological turn leads farther into the journey, departing from its constructionist antecedent. The ontological turn is fundamentally about “what is not captured in discourse”; it also captures materiality—specifically, environments, objects and artifacts—and moves us from language/representation to “embodied engagements and the entanglements of material *and* discursive realities” (Zembylas, 2017: 1401, 1409, italics added; Barad, 2007; Feely, 2016; Todd, 2016). Bodies and objects are taken as subjects of study set against their symbolic meaning, bringing together the “conceptual” and “empirical level” simultaneously and as interwoven (Zembylas, 2017: 1402). The ontological turn does not do away with discourse but rather embraces it while also taking account of events, practice, and the material; thereby, it is not a break, but a continuity (Barad, 2007; Feely, 2016). Rather than in intentionality, privacy, and individuality of meaning and human experience, the world as we know it in the ontological turn exists instead as assemblages (Delanda, 2016), entanglements (Barad, 2007), ‘sila’^3^ (Todd, 2016), relationalities (Zembylas, 2017), comprising both the human and the nonhuman.

## Coronavirus lessons of ontology: from the migrant farmworker

Ontology is usually a matter of philosophical and conceptual abstractions. But our current moment—the migrant farmworker in the time of Covid-19—offers an opportune occasion or case for reflecting on the ontological turn as evidenced in the real world. This empiricity offers us a moment to “recalibrate the level at which analysis takes place,” (Zembylas, 2017), a moment all the more relevant since the matter at hand is *essentiality*, which lies at the core of ontology. Having background for our ontological journey and its recent turn (discussed above) is needed in the first place to better situate what is at hand (detailed next) and envision realities moving forward (discussed in closing).

Returning to the first sentence in this paper, we look again for lessons of ontology.^4^ It has perhaps become impossible to dismiss how U.S. political discourse has increasingly vilified migrants, especially undocumented migrants. Through a constructionist ontological lens, this discourse subjectifies the migrant, forging realities that result in her detention, separation from her family, suffering, and even death. But with the pandemic, the federal government was cornered into a reversal: a different representation of the migrant farmworker as essential. That shift was sudden, occurring as quickly as the new coronavirus spread. And the shift could not be more opposing, from the *illegal* worker who was deportable to the *essential* worker who was heroic (Heroes’ Act, 2020)^5^. “For many workers, the fact that they are now considered both illegal and essential is an irony that is not lost on them, nor is it for employers who have long had to navigate a legal thicket to maintain a work force in the fields” (Jordan, 2020). The suddenness of the discursive shift and its radical and literal contradiction in terms render the powers of discourse (and of the federal government as speaker) palpable and unmistakable.

Yet, this discursive rendering seems unsatisfying and puzzling because the migrant farmworker has always been essential; here is our first lesson on ontology: *post-representation.* Migrant labor has always been a cornerstone of the U.S. agriculture industry, with or without the pandemic, whatever the discourse. The discursive world around the migrant farmworker shifted, but neither the migrant herself nor the food industry she sustains shifted. This laboring–physical, embodied, and material– is often not fully understood via a discourse lens as they focus on the construction of such discourse to reveal its violence. And this disjuncture—between the discourse/recognition of labor and the materiality/embodiment of laboring—is at the heart of the ontological turn.

Accordingly, the rest of Jordan’s *New York Times* article expands the analysis beyond the focus on discourse in the lead. A post-representational^6^ take on the migrant farmworker, one along the ontological turn, is an undertaking that approaches wholeness in that it also accounts for materiality (Feely, 2016). This is not a move away from discourse; on the contrary, discourse has much to say (Foucault, 1984), especially about things that cannot be said in the first place or said at all. Indeed, analysis of discourse often reveals what is missing. However, attending not only to public and media discourse on the migrant farmworker, which is concerned with the social and the cultural, can open up a way into the economics of im/migration.

Getting to economics warrants a look at the fruit that manifests from and with the discourse, our second lesson on ontology: *a focus on objects and artifacts.* This, Jordan did do, with citrus in California, Georgia peaches, and Washington apples. While the literal object was subtle in Jordan’s article, we can perhaps see how the fruit-as-product is central in analyzing the representation-materiality dilemma posed at the outset. Fruit, as material (Introna, 2019; Zembylas, 2017), allows an entry into seeing, and tasting, the co-constitution of the social (farmworker, employer, consumer, food culture) and the material (fruit, profit, laboring bodies, farmland). It allows for the entwining of the power of discourse with the presence of the tangible, enabling us to understand the profound contradictions between a social structure that doesn’t want the migrant and a capitalist economy that needs her. Further, as Jordan reports, policies on detention and deportation and policies on pandemic relief aid are of relevance: While they use language and enact meaning, policies, too, are very material, yielding the felt impacts of being placed behind bars and having income, respectively.

## The Co-Constitution of the migrant farmworker and Us as consumers

Jordan’s article continues with labor unions, activists and advocates, farm owners or employers, government policies and policing, and the migrant herself in Ms. Silva, illustrating the relationality of things, the non/human assemblage of actors, institutions, infrastructures, environments and industries that are necessarily connected to the migrant farmworker. Here, we come to our final lessons of ontology: *posthumanism and the politicizing of ontology.* For Badriotti, “becoming posthuman is a *process* of redefining one’s sense of attachment and connection to a *shared* world,” (2016: 25, italics added). Indeed, for indigenous thinkers, the environment has long been “a *common* organizing force” (Todd, 2016, italics added). Extending our gaze outward binds us to our relationality. Like an earthquake, an ontological shift in one aspect triggers shifts and tremors in another, given relationality as bedrock. The migrant’s illegality-essentiality as discursive ambivalence and her fruit and embodied labor as material now couple into a fuller ontology, insofar as together they link the migrant to industry, to capitalist production-consumption and, finally, to our dining tables.

A shift in the migrant-other necessarily entails a shift in the consumer-us and the worlds we inhabit. Shifting the gaze inward as consumers can be a political act, an action designed to attain a purpose through the use of power. If “the real” is relational and processual or dynamic, and if we-as-consumers are on the other side of the migrant farmworker in this relationship and dynamic, then we compose the real and we can make the change happen. Possibility and potential constitute ontology. Amidst the pandemic, during a migrant farmworker advocacy videoconference (Zocalo Public Square, 2020), food activist-farmer Nikiko Masumoto called for narrowing the gap between consumers and food producers, and building relationships between these two communities, as we transition into a “new normal.” Masumoto said, “If only we can open up this moment, so that we can find out what kind of world we can emerge.”

## Notes

1 The concept of “essentiality” as used in this paper should be differentiated from “essentialism” and “essence” as based on biology and denoting unchangeability (see Feely, 2016, for elaboration).

2 Meat industry workers, also primarily immigrants, are also implicated in this, as COVID-19 turned meat factories across the country into hotspots and threatened the supply of meat during the pandemic (Laughland and Holpuch, 2020).

3 *Sila* is an Inuit concept denoting the ontological turn and post-humanism, based on critical indigenous scholarship (Todd, 2016). *Sila* “links environment to knowledge” (Martin 2012 in Todd, 2016: 5) and “is bound with life, with climate, with knowing, and with the very existence of being(s)” (Todd, 2016: 5).

4 The four lessons of ontology in this article were drawn from Zembylas’s four “key themes of the ontological turn” (2017: 1404).

5 This pandemic relief aid, passed in April 2020, provided resources for essential workers, but migrant farmworkers were excluded from all aid although the government certified them as essential workers in other legal documents.

6 Zembylas (2017) uses the term *non*-representation, but I use *post*-representation, because I focus on the ontological turn as a complementarity continuity rather than a break from representation, post-structuralism, or discursivity, following Feely (2016) and Barad (2007).
